# Correction to “miR‐203 inhibits augmented proliferation and metastasis of hepatocellular carcinoma residual in the promoted regenerating liver”

**DOI:** 10.1111/cas.16361

**Published:** 2024-10-30

Zheng XB, Chen XB, Xu LL, Zhang M, Feng L, Yi PS, Tang JW, Xu MQ. miR‐203 inhibits augmented proliferation and metastasis of hepatocellular carcinoma residual in the promoted regenerating liver. Cancer Sci 2017; 108 (3): 338–346.

In Figure 3A, an incorrect image was used to show Ki67 expression in HCC tissue of the PH group. Furthermore, the Tubulin alpha western blot was incorrectly labeled in figure 5B.

The correct images in Figure 3A are shown below.

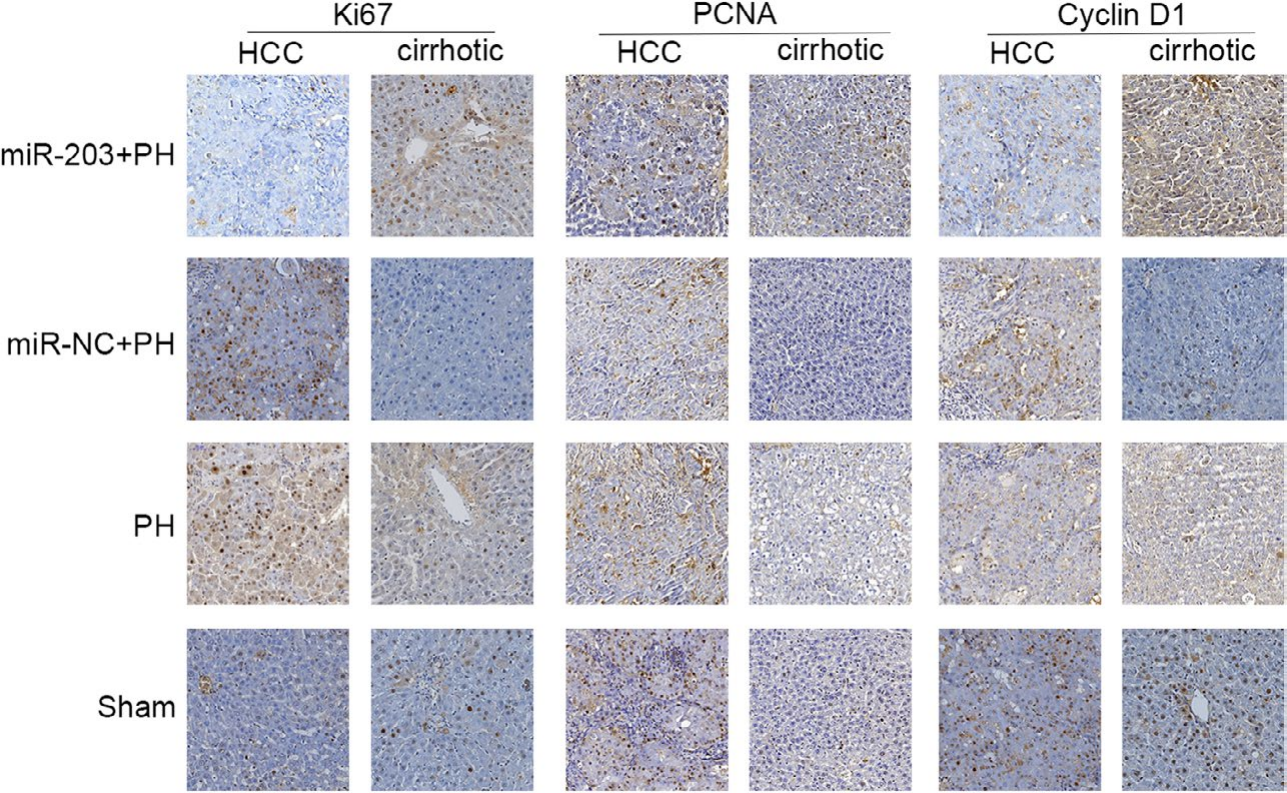

The correct images in Figure 5B are shown below.

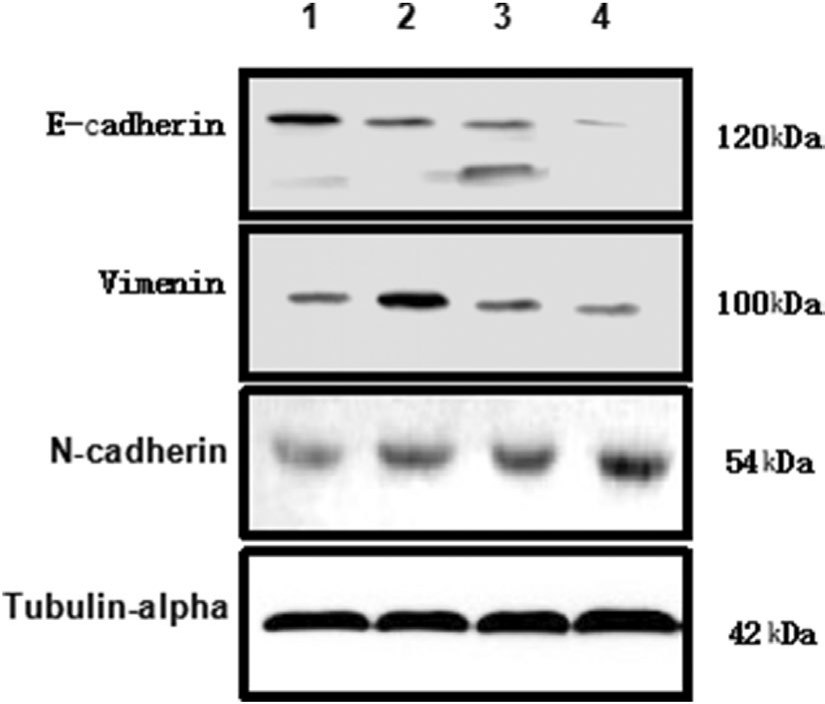

The authors apologize for these errors.

